# Evaluation of long-term welfare initiatives on working equid welfare and social transmission of knowledge in Mexico

**DOI:** 10.1371/journal.pone.0251002

**Published:** 2021-05-04

**Authors:** Emily Haddy, Faith Burden, José Antonio Fernando-Martínez, Dafne Legaria-Ramírez, Zoe Raw, Julia Brown, Juliane Kaminski, Leanne Proops

**Affiliations:** 1 Department of Psychology, Centre for Comparative and Evolutionary Psychology, University of Portsmouth, Portsmouth, United Kingdom; 2 The Donkey Sanctuary, Sidmouth, Devon, United Kingdom; 3 Facultad de Medicina Veterinaria y Zootecnia, Programma Donkey Sanctuary–Universidad Nacional Autonoma de Mexico, Universidad Nacional Autonoma de Mexico, D.F. Mexico, Mexico; 4 School of the Environment, Geography & Geosciences, University of Portsmouth, Portsmouth, United Kingdom; Massey University, NEW ZEALAND

## Abstract

Working equids play an essential role in supporting livelihoods, providing resilience and income security to people around the world, yet their welfare is often poor. Consequently, animal welfare focussed NGOs employ a range of initiatives aimed at improving standards of working equid welfare. However, there is debate surrounding the efficacy of welfare initiatives utilised and long term monitoring and evaluation of initiatives is rarely undertaken. This study compares equid welfare and the social transmission of welfare information across Mexican communities that had previously received differing intervention histories (veterinary treatment plus educational initiatives, veterinary treatment only and control communities) in order to assess their efficacy. Indicators of equid welfare were assessed using the Equid Assessment Research and Scoping tool and included body condition score, skin alterations, lameness, general health status and reaction to observer approach. Owners were interviewed about their involvement in previous welfare initiatives, beliefs regarding equid emotions and pain, and the social transmission of welfare knowledge, including whether they ask advice about their equid or discuss its health with others and whether there is a specific individual that they consider to be ‘good with equids’ in their community. In total 266 owners were interviewed from 25 communities across three states. Better welfare (specifically body condition and skin alteration scores) was seen in communities where a history of combined free veterinary treatment and educational initiatives had taken place compared to those that had only received veterinary treatment or control communities. The social transfer of welfare knowledge was also higher in these communities, suggesting that the discussion and transfer of equid welfare advice within communities can act as a mechanism to disseminate good welfare practices more widely. Our results suggest that using a combined approach may enhance the success of welfare initiatives, a finding that may impact future NGO programming.

## Introduction

There are an estimated 100 million equids in low to middle income countries [1]; the majority of which are working animals. Globally, they play an essential role in supporting livelihoods, generating incomes and supporting families [2–4]. They also provide a social lifeline, providing resilience, security and income diversification to many groups including women, marginalised communities and those in extreme poverty [2,5,6]. As a consequence of reduced access to basic resources, despite their importance, working equids often suffer from poor welfare [7]. Research from many countries has demonstrated high levels of welfare problems such as wounds, lameness, poor body condition, and environmental stress from working in extreme conditions [7–11]. These reduce an animal’s productivity, thereby limiting the income generation and support they can provide for those that rely upon them [9,12].

In response to these concerns, a range of animal welfare focussed non-governmental organisations (NGOs) strive to improve standards of working equid welfare. Different models and approaches have been employed by different NGOs over time, and vary depending upon the size and philosophy of the organisation, the areas of the world in which they work and their funding sources [13]. These initiatives include the use of participatory methods [14], educational programmes for school children [15], advocacy [16], the provision of access to free veterinary treatment [10,17–19], providing technical training and skills in fields such as farriery and saddlery to individuals in equid owning communities [20,21], and initiatives that target specific aspects of welfare such as handling and behaviour [22] or lameness [23]. There is debate surrounding the efficacy of the range of welfare initiatives that are implemented. Specifically, doubts exist about whether the initiatives significantly change the way owners manage their animals [24], whether the effects of welfare initiatives last in the long term [25], and which of the models are the most effective in achieving sustained welfare outcomes. In the past, the provision of veterinary care was the most common approach, with a range of services offered from preventative care to emergency treatment [13]. However, there is concern that a service based approach is unsustainable in the long term. Veterinary treatment is often offered for a short period of time with specialist equipment and drugs transported for this purpose but after withdrawal, lack of existing animal health infrastructure and inability to follow up cases can prevent long term welfare improvement [4]. It has also been highlighted that purely service based approaches are likely to be treating the symptoms rather than the root causes of welfare problems [20,24]. The creation of dependency on free service provision is also a concern when services are likely to, at some point, be withdrawn [13]. This has led to a focus on proactive (those that focus on the long-term prevention of welfare issues) rather than reactive interventions.

One method used to try to improve the sustainability of an initiative is to introduce specific knowledge or a set of skills to an equid owning community. This information can then be utilised by owners or handlers with the hope that it is retained within the community in the future and hence reliance on external organisations is reduced. Discussion between individuals provides an avenue for equid welfare knowledge and skills to be introduced and distributed within a target community; this was termed ‘community learning’ by Rodríguez Rodas and Pérez [26] and involved peer-to-peer transfer of information within a social network. The combination of information transfer and learning through social influence has also been described as particularly effective in human healthcare interventions [27]. The model has been utilised in the training of equid owner change agents in Ethiopia, with the premise that change agents will transfer their skills and knowledge to peers and this will more widely ‘trickle down’ to other equid owners [28]. Trickle down of knowledge has also been highlighted as a common assumption in the international development field [29]. By this principle, the assumption is that the higher the degree of social transfer of this information (be it discussion of best working practices or of how to effectively treat common health problems), the more people within communities will be reached by NGO initiatives, even if that person did not directly attend a workshop or clinic. Despite the social transfer potential for the retention of knowledge and skills within a community [30], there have been very few studies examining the persistence of working equid welfare knowledge after NGO delivery and withdrawal. Similarly there have been very few studies which have reflected upon how this type of knowledge is transferred within a community. Monitoring, evaluation (and subsequent learning) is vital to informing the types of initiatives that are most effective for welfare change and knowledge persistence for communities. However, the general lack of monitoring and evaluation of working equid welfare initiatives has been highlighted [13]. In reality effective monitoring and evaluation is not always easily achievable; the impact of human behaviour change can take a long time to be realised [31] and after the implementation of a welfare initiative it may take years before improvements in equid welfare are seen–well beyond the funding cycle of the NGO [32]. Previously, the presence of services and indicators which recorded process, for example number of animals treated or number of service providers trained, tended to be used, rather than outcomes measures that directly assess improvements in welfare [13,33,34]. However it is increasingly recognised that the use of suitable outcome based indicators, which address the issues that the intervention was designed to tackle, and the extent to which improvements represent optimal use of resources, are necessary [13,35]. Some studies have therefore used animal based welfare indicators to address this [18,33], others use mainly qualitative reporting on the views of people in communities in order to reflect behaviour change or knowledge gains [14,17].

In this study we focussed on evaluating the long term effects on equid welfare of the provision of free veterinary treatment and two types of educational initiatives, farriery courses and handling workshops. The study is novel in its long term focus across multiple initiatives types and contributes to knowledge of an under researched area. Two hundred and sixty six owner interviews and equid welfare assessments were conducted in Mexican communities that had previously experienced varying levels of interventions implemented by NGO The Donkey Sanctuary (no intervention, vet clinic alone or vet clinic plus educational workshops). Information on the social knowledge transfer of equid management practices was also collected across communities with differing intervention histories.

## Methods

### Study locations

**Fig 1:**
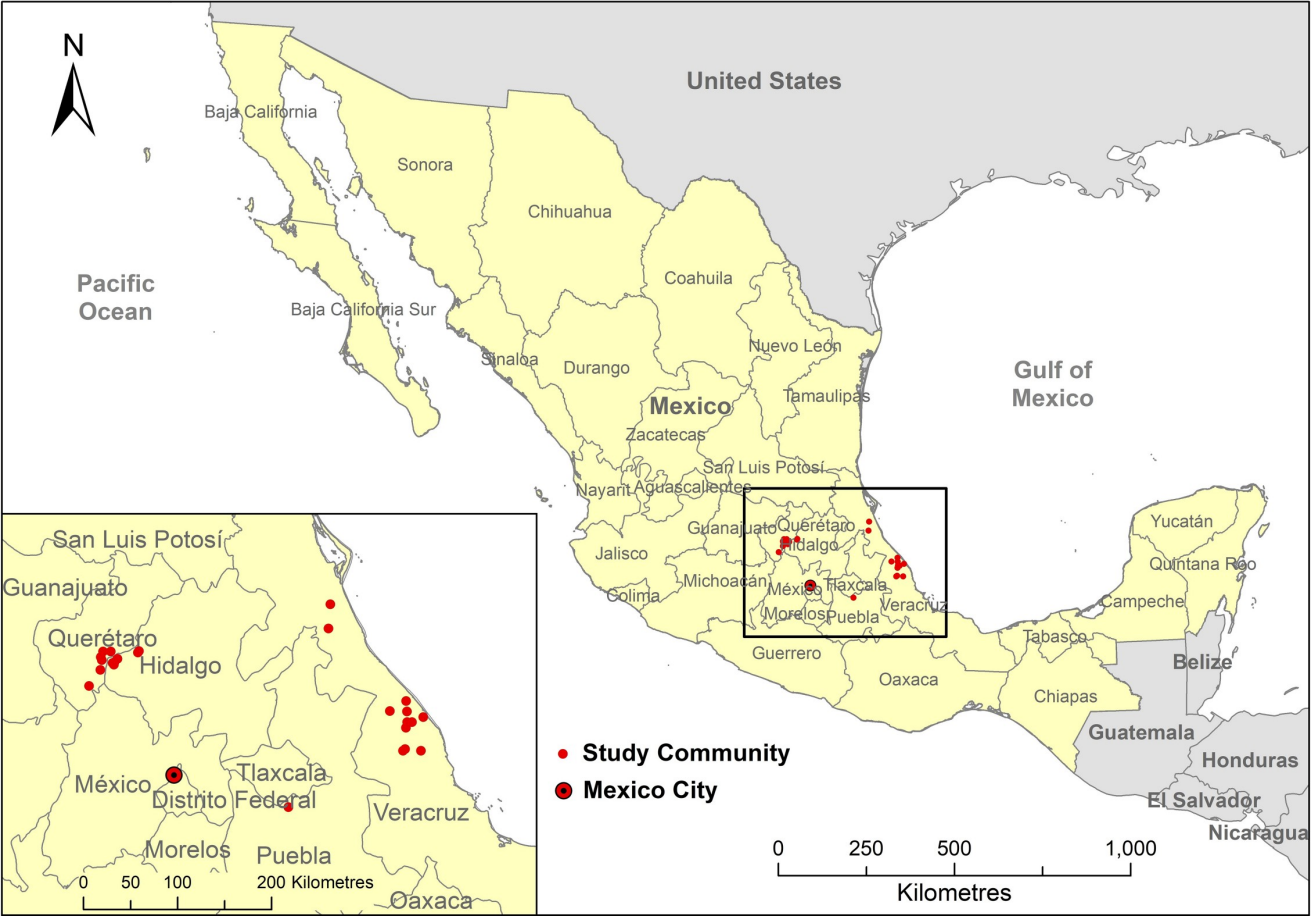
Map showing the location of the study communities within Mexico. Country data source: Diva-GIS.

Data were collected from three states in Mexico (Veracruz, Querétaro, Puebla); all communities were rural villages (Fig 1). Communities were selected due to their previous participation in welfare initiatives run by NGO The Donkey Sanctuary. Control communities in the same geographical area that had not received any type of welfare initiative, but were known to use working equids, were recruited for comparison. The three states differ in their climatic conditions: Veracruz is classified according to the Köppen Climate Classification [36] as a tropical savanna climate. Querétaro has a classification of a hot semi-arid climate and Puebla is a subtropical highland climate, both of these climates result in less abundant and lower quality forage in comparison to Veracruz. In total 25 communities were visited over a period of two years, from January to April 2019 and from January to March 2020.

### Classification of villages by intervention type

Communities were classified into categories of high, low or no intervention based on the type of initiatives they had previously received. Any community that had received educational handling workshops or farriery courses alongside free veterinary clinics were classified as high intervention, communities who had received only free veterinary clinics were classified as low intervention and those who had no NGO involvement acted as control communities (no intervention). Veterinary clinics had been run either annually or biannually for at least 8 years, with the last clinic having taken place during the time period of data collection for all communities except one whose last clinic was 4 years ago. All educational initiatives had taken place two to five years before the period of data collection with the exception of one which had taken place 10 years ago.

### Study population

The study was approved by both the University of Portsmouth’s Ethics Committee (reference number SFEC 2019–112), and the University of Portsmouth’s Animal Welfare Ethics Research Board (reference number 1219E). Approval for the research to take place in each village was granted from relevant authorities (local government or members of the livestock association) and oral consent was obtained for all participants. All owners and handlers also gave informed consent for their animals’ inclusion in the study. In total 266 equid owners and handlers participated in the study, 48 women and 218 men with ages (excluding two participants who chose not to disclose their age) ranging between 16 and 84 (mean = 47.3, s.d. = 16.5 years). Participants were interviewed from 25 communities across 3 states: Veracruz (n = 150), Querétaro (n = 60) and Puebla (n = 56).

### Study animals

A total of 121 donkeys (females = 40, stallions = 61, geldings = 20), 15 mules (females = 7, stallions = 3, geldings = 6) and 130 horses (females = 46, stallions = 29, geldings = 55) were assessed. The average age of the equids whose ages were known (n = 250) was 8.3 years (min = 1, max = 33, SD = 5.8 years).

### Materials

The Equid Assessment Research and Scoping (EARS) tool [37], developed by UK-based NGO The Donkey Sanctuary was used to assess equid welfare and management practices. EARS is a comprehensive tool utilising a variety of validated welfare indicators, identified as having a substantial influence on equid welfare. The EARS tool is designed to be able to assess the welfare of all working equid species and allows standardised comparisons of welfare assessments across the diverse contexts in which equids are found; in this study between a range of working roles, communities and regions. The EARS tool allows the creation of protocols for particular assessment aims or research questions. A protocol was created for this study consisting of questions relevant to the study context which included sections on working and management practices; and for the equid welfare assessment, subsections on behaviour, body condition, the skin system, the musculoskeletal system, and health status. Once created, the protocol was put into Open Data Kit (ODK) Collect software [38] on an android tablet for ease of data collection in the field. The results of assessments were available to download as Microsoft Excel files. All welfare assessments were completed by E.H., who was familiar with the EARS protocol and had been checked for inter-observer reliability against other trained assessors prior to the onset of this study.

### Procedure

The researcher (E.H.) accompanied staff and veterinary students from the Donkey Sanctuary Mexico and the National Autonomous University of Mexico (UNAM) during their work in high and low intervention communities. In no intervention communities (those that fall outside of the Donkey Sanctuary Mexico’s target areas) the researcher and a translator worked alone. Animals were sampled for assessment from a range of situations: some were scheduled for castration, some attended free mobile clinics (providing worming, health checks and treatment for ill or injured animals) and some were recruited through random door-to-door sampling in the selected communities. Opportunity sampling of working equid owners was used in order to maximise sample size in each situation. Owners were approached by the researcher and a translator, who was a fluent native speaker. The study was explained to potential participants, and verbal consent was obtained (this was felt to be more appropriate than written consent due to potential variation in participant literacy levels). For those participants who owned multiple animals, only one equid per owner was chosen by random number selection and assessed. Firstly, whilst the owner held the equid, the short behavioural and physical welfare assessment from the EARS tool [37] was carried out. The body condition score (measured on a 5 point scale), skin alterations (for analysis grouped into 3 categories: serious alterations including large alterations and wounds or scars caused by tack and/or beating, small alterations including small wounds or scars and those resulting from accidents or fights with other animals, no alterations), lameness (assessed visually and categorised on a 3 point scale: unable to walk, lame but able to walk, no lameness), general health status (scored on a 3 point scale: good, fair or poor based upon cumulative analysis of welfare markers) and reaction to observer approach (friendly, neutral, avoidant, agonistic) were recorded by the researcher. Secondly, a structured interview of 10 questions was conducted with owners. The researcher asked each owner whether they had participated in any of the previously run welfare initiatives, and if so when these occurred. Owners were also asked the primary role of their equid and whether they believed that their equid could feel emotions and pain (measured as yes, no or unsure). The final questions related to the social transmission of welfare and handling knowledge within their community. Owners were asked whether they ask advice about their equid (and if so who they ask), whether they discuss the health of their equid with others and whether there is a particular person within their community that they consider to be ‘good with equids’ (and if so why). The duration of interviews was on average between 15 and 40 minutes. Responses were translated to English directly throughout the interview by the translator and audio recorded for later verification and content transcription of qualitative data.

#### Statistical analysis

Data from the structured interview were used to generate quantitative data for statistical analysis. Descriptive statistics were calculated for the population. The data were considered to be unsuitable for multivariate modelling due to the high degree of covariation between species, working role and location (state). In the study regions, clearly defined relationships existed between equid species and working role with most donkeys used for packing and most horses for riding [39]. The presence of equid species also varied by state with the proportion of donkeys assessed far higher in Puebla compared to the other states. Instead, a series of Kruskal-Wallis tests (with Bonferroni correction for multiple tests) were used to assess differences in welfare markers (body condition score, skin alteration score and general health score) based on location (state) and intervention level independently. Kruskal-Wallis tests were also used to assess differences in owner attitude across intervention levels. Mann Whitney U tests were used to assess differences in welfare markers based on role (riding or packing). As modelling was not possible, in order to try to disentangle the effects of location, role and intervention and explore the effects of intervention in isolation, an additional analysis was conducted of riding equids in Veracruz alone. Veracruz was chosen because of the large sample size (n = 84) and analysis of this single area allowed for the effect of climate to be mitigated as the climatic conditions in all Veracruz locations were tropical [36] with abundant available forage. Importantly a substantial number of communities from all three intervention categories (high, medium, low) were also included in the Veracruz sample (which was not possible for other states). Chi-square tests (3x2) with post hoc pairwise comparisons adjusted for multiple testing [40] were used to test for differences in the social transfer of equid welfare information (whether owners ask others in their local area for equid advice, whether they discuss the heath of their equid with others and whether there is a particular person in their community that they consider to be good with equids) based on intervention level. Analyses were performed using SPSS Version 26.0 [41].

## Results

### The relationship between intervention level and welfare

**Fig 2:**
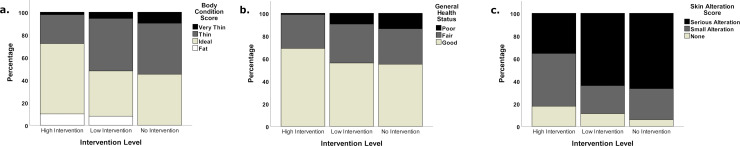
Stacked bar charts showing the percentage distribution of body condition scores, general health status scores and skin alteration scores across intervention levels.

Body Condition Score: Overall 5% (n = 14) of equids were found to be very thin, 39% (n = 104) were thin to moderate, 49% (n = 129) were ideal and 7% (n = 19) were fat. The data collection period did not coincide with the peak agricultural workload and as such, average body condition may be higher than when in full work. There were significant differences in body condition score based on intervention level (Σ^2^ (2) = 16.05, *p* < 0.001), with higher body condition scores seen in high intervention communities in comparison to both low intervention (Σ^2^ (1) = 31.69, *p* = 0.003) and no intervention communities (Σ^2^ (1) = 43.47, *p* = 0.001), but no difference between low intervention and no intervention communities (Σ^2^ (1) = 11.78, *p* = 0.93) (Fig 2A).

#### General health status

Overall, 60% (n = 160) of equids were classified as being in good health, 32% (n = 86) were in fair health and 8% (n = 20) in poor health. There were significant differences in general health status based on intervention level (Σ^2^ (2) = 6.25, *p* = 0.04), however after Bonferroni correction for multiple tests, there were no significant pairwise differences between intervention levels (Fig 2B).

#### Skin alterations

Overall, 55% (n = 146) of equids showed serious skin alterations, 33% (n = 87) showed small skin alterations and 12% (n = 33) did not show any skin alterations. The most frequent cause of skin alterations was tack (saddle, girth and bridle or noseband), and was seen in 54% (n = 143) of equids, alterations caused by insects were observed in 13% (n = 35) of equids and alterations caused by neck tethering or hobbling were observed in 7% (n = 19) of equids. There were significant differences in skin alterations based on intervention level (Σ^2^ (2) = 19.40, *p* < 0.001), with a lower incidence of skin alterations seen in high intervention communities in comparison to both low intervention (Σ^2^ (1) = 37.08, *p* < 0.001) and no intervention communities (Σ^2^ (1) = 43.38, *p* = 0.001), but no difference between low intervention and no intervention communities (Σ^2^ (1) = 6.30, *p* > 0.99) (Fig 2C).

#### Lameness

Visual signs of lameness were observed in 8% (n = 20) equids when moved by their owner. There was no significant difference in lameness across intervention levels (Σ^2^ (2) = 5.02, *p* = 0.08).

#### Behaviour

Overall responses to observer approach by equids were: friendly 62% (n = 165), neutral 10% (n = 27), avoidant 27% (n = 72) and agonistic 1% (n = 2). Responses to walking down the side of equids were: positive 40% (n = 107), neutral 47% (n = 124) and negative 13% (n = 35). A tail tuck (a sign of fear) was only observed in 3% (n = 7) of donkeys and mules and chin contact was accepted by 81% (n = 215) of equids. There was no significant difference in response to observer approach (Σ^2^ (2) = 1.46, *p* = 0.48), response to walking down the side of the equid (Σ^2^ (2) = 4.35, *p* = 0.11), or acceptance of chin contact (Σ^2^ (2) = 2.23, *p* = 0.33) across intervention levels.

### The relationship between location and role and welfare

The primary roles of the equids assessed were as follows: 43% (n = 115) riding, 41% (n = 110) packing 7% (n = 19) agroforestry, 4% (n = 11) sport and 4% (n = 11) other. Working roles were clearly defined by species with 80% (n = 97) of donkeys used for carrying goods by pack, 75% (n = 98) of horses used for riding and 53% (n = 8) of mules used for agroforestry. Distribution of species and (subsequently working role) also differed by state, 89% (n = 50) of equids assessed in the state of Puebla were donkeys whereas in Veracruz and Querétaro respectively, 60% (n = 90) and 63% (n = 38) of equids assessed were horses.

There were significant differences in our three main indices of welfare based on location (body condition score: Σ^2^ (2) = 20.26, *p* < 0.001; general health status: (Σ^2^ (2) = 26.91, *p* < 0.001; skin alterations: Σ^2^ (2) = 27.46, *p* < 0.001). Across all three measures, scores were significantly lower in Puebla when compared to Querétaro (body condition score: Σ^2^ (1) = 57.91, *p* < 0.001; general health status: Σ^2^ (1) = 59.83, *p* < 0.001; skin alterations: Σ^2^ (1) = 59.36, *p* < 0.001) and Veracruz (body condition score: Σ^2^ (1) = 35.30, *p* = 0.004; general health status: Σ^2^ (1) = 46.34, *p* < 0.001; skin alterations: Σ^2^ (1) = 51.24, *p* < 0.001), but there were no differences between Querétaro and Veracruz (body condition score: Σ^2^ (1) = 22.61, *p* = 0.1; general health status: Σ^2^ (1) = 13.49, *p* = 0.55; skin alterations: Σ^2^ (1) = 8.12, *p* > 0.99). There were significant differences in body condition score based on role, packing animals had significantly lower body condition scores (U = 4816, *p* = 0.001), poorer general health status (U = 7588, *p* = 0.003) and more skin alterations (U = 4927, *p* = 0.001) compared to riding animals.

#### Riding equids in Veracruz

**Fig 3:**
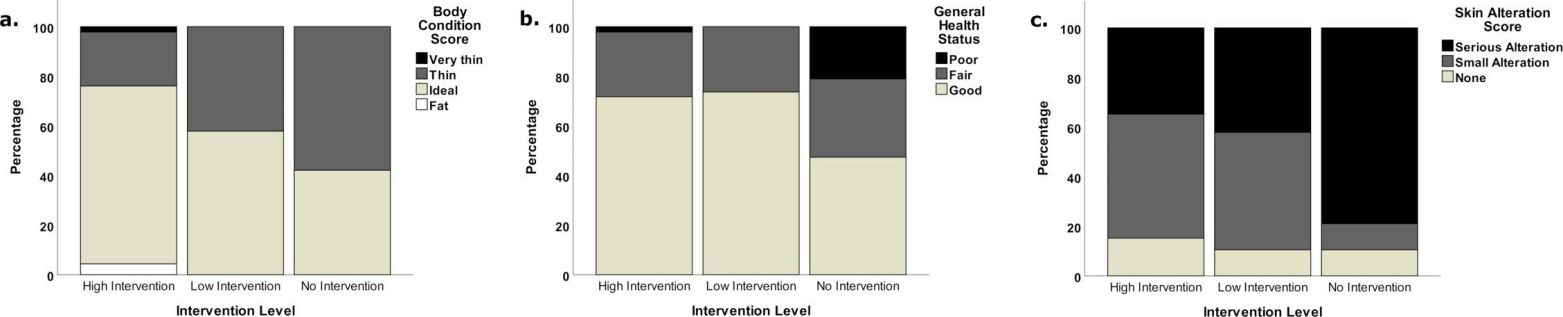
Stacked bar charts showing the percentage distribution of body condition scores, general health status scores and skin alteration scores of riding equids in veracruz across intervention levels.

To disentangle the effect of intervention from the effects of both location and role, an additional analysis of riding animals in Veracruz only was conducted. When analysis was conducted on this reduced sample, the significant difference in body condition score based on intervention level remained (Σ^2^ (2) = 7.22, *p* = 0.03), with higher body condition scores seen in high intervention communities in comparison to no intervention communities (Σ^2^ (1) = 14.61, *p* = 0.03), but no difference between high and low intervention communities (Σ^2^ (1) = 8.22, *p* = 0.44) nor low and no intervention communities (Σ^2^ (1) = 6.40, *p* > 0.99) (Fig 3A). There was a trend towards general health status being different across intervention levels (with better health status seen in higher intervention communities (Σ^2^ (2) = 5.77, *p* = 0.06)) (Fig 3B). There was also a significant difference in skin alterations based on intervention level (Σ^2^ (2) = 8.07, *p* = 0.02), with a lower incidence of skin alterations seen in high intervention communities in comparison to no intervention communities (Σ^2^ (1) = 17.18, *p* = 0.01), but no difference between high and low intervention communities (Σ^2^ (1) = 3.73, *p* > 0.99), nor low and no intervention communities (Σ^2^ (1) = 13.45, *p* = 0.19) (Fig 3C).

### The relationship between intervention level and owner attitudes to equid sentience

Overall, 12% (n = 32) of owners were unsure or did not believe that their equid could feel emotions and 5% (n = 14) were unsure or did not believe that their equid could feel pain. There was a significant difference in owner belief that their equid could feel emotions based on intervention level (Σ^2^ (2) = 7.44, *p* = 0.02), with a significantly higher proportion of owners believing that their equid could feel emotions in high intervention communities in comparison to low intervention communities (Σ^2^ (1) = 16.02, *p* = 0.02), but no difference between high and no intervention communities (Σ^2^ (1) = 5.65, *p* > 0.99), nor low and no intervention communities (Σ^2^ (1) = 10.37, *p* = 0.45). Similarly, there was a significant difference in owner belief that their equid could feel pain based on intervention level (Σ^2^ (2) = 6.41, *p* = 0.04), with a significantly higher proportion of owners believing that their equid could feel pain in high intervention communities in comparison to low intervention communities (Σ^2^ (1) = 10.23, *p* = 0.04), but no difference between high and no intervention communities (Σ^2^ (1) = 3.74, *p* > 0.99), nor low and no intervention communities (Σ^2^ (1) = 6.49, *p* = 0.57).

### Social transfer of welfare information

**Fig 4:**
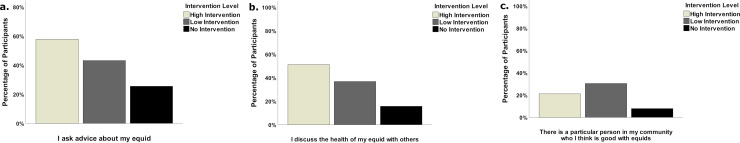
Bar graphs showing the percentages of participants across intervention levels who a) ask for advice about their equid, b) discuss the health of their equid with others, c) think that there is a particular individual in their community who is good with equids.

Overall, 45% (n = 119) of participants responded that they did ask advice about their equid from others. Of these participants, the most commonly cited sources of advice were vets (n = 43), family (n = 30), friends or neighbours (n = 20) and The Donkey Sanctuary staff (n = 10). There was a significant difference in whether participants asked advice about their equid according to intervention level, Σ^2^ (2) = 13.95, *p* = 0.001 (Fig 4A). Compared to the expected chi square values (even across all levels), high intervention communities asked for advice significantly more than expected (*p* = 0.002), low intervention communities asked for advice at rates that were not significantly different from what would be expected (*p* = 0.7) and no intervention communities asked for advice significantly less than expected (*p* = 0.001).

In total, 38% (n = 100) of participants responded that they discussed the health of their equid with others. There was again a significant difference in whether participants discussed their equid’s health according to intervention level, Σ^2^ (2) = 17.48, *p* < 0.001 (Fig 4B). Compared to the expected chi square values, high intervention communities discussed the health of their equid significantly more than expected (*p* = 0.001), low intervention communities at rates that were not significantly different from what would be expected (*p* = 0.8) and no intervention communities discussed equid health significantly less than expected (*p* < 0.001).

Within their community, 23% (n = 61) of participants thought that there was a particular person who was good with equids. Participants considered these named individuals to be ‘good with equids’ for a variety of reasons including being experienced, treating their animals well, having attended educational workshops, knowing many people so gaining lots of advice, giving advice and possessing knowledge and skills (handling, training and breaking, farriery, knowledge of diet and of medicine were specifically mentioned). Of the individuals named as being ‘good with equids’ 34% (n = 21) had previously attended an educational handling or farriery workshop. There was a significant difference in whether participants identified an individual in the community as being particularly good with equids according to intervention level, Σ^2^ (2) = 10.68, *p* = 0.005 (Fig 4C). Compared to the expected chi square values, high intervention communities identified individuals at rates that were not significantly different from what would be expected (*p* = 0.4), low intervention communities identified individuals significantly more than expected (*p* = 0.005) and no intervention communities identified individuals significantly less than expected (*p* = 0.01).

## Discussion

The differences in welfare seen across intervention levels suggests that the initiatives put in place, in particular the combined approach of educational initiatives and free veterinary clinics, have been making a difference in improving welfare in the target communities, specifically body condition score and skin alterations. Behavioural indicators and lameness did not co-vary with intervention level. Poor body condition and wounds were the most common welfare problems observed across all study locations and this suggests that the interventions put in place have been effectively targeting these most prevalent issues. Previous research in the study area has demonstrated that role, location and species have an effect on equid welfare [42] although the natural covariance of these factors means that it is not possible to isolate their relative effects. In order to ensure that the influence of location and role were not confounding the effect of intervention level on welfare, a separate analysis of riding equids in the state of Veracruz only was conducted. Results confirm that the differences seen in body condition scores and skin alterations are linked to the initiatives implemented and are not simply a product of environment or working role. The social transfer of welfare information was highest in high intervention communities; in particular equid owners were more likely to ask others for advice and discuss the health of their equid with others when compared to low and no intervention communities whereas individuals considered to be good with equids were identified more than expected in low intervention communities. A higher proportion of owners from high intervention communities compared to low intervention groups also believed that their equids could feel emotions and pain; however these levels were similar to the no intervention group.

High intervention communities (those with a combination of educational training and free veterinary treatment) showed consistently better levels of welfare compared to low and no intervention communities. The comparisons between low intervention and no intervention groups were not significant, potentially indicating that the educational training, unique to high intervention communities, is the primary factor influencing the better levels of welfare seen in these areas. However, despite the lack of statistical significance between welfare markers in low and no intervention communities, those in low intervention communities were always intermediate between those in the high intervention and no intervention communities. This could indicate that statistical power or effects sizes were not large enough to show smaller pairwise differences between the low and no intervention levels. In this case, it would suggest that a combination approach may be proving to be the most effective in terms of improving welfare within the target communities, although further research is needed to clarify this relationship. Combined intervention strategies have been recommended to increase intervention efficacy both within equid welfare [43] and in other fields including health behaviour and education [44,45]. Free veterinary clinics may be especially useful for owners who cannot afford or do not have access to veterinary treatment for their animal locally [17]. The information given by vets to owners during examination, for example information on wound hygiene and the necessity of regularly cleaning hooves, can be put into practice by owners to improve their animal’s welfare beyond the provision of free treatment. It would be useful for future studies to include comparison areas in which educational workshops or training courses were the only intervention, this would allow for the influence of these educational components alone to be compared.

The skills targeted in the educational initiatives (in this case handling and farriery) may be especially relevant to improving welfare and attitudes in the study communities. Handling workshops aim to improve the communication and working relationship between an owner and equid. This may reduce the use of harsh control methods which have been linked to wound prevalence [46] and is particularly pertinent to the study communities where tack was the main cause of skin alterations observed. How an equid perceives its environment and how to read the behavioural cues associated with different equid emotions (such as fear) form part of the handling and farriery courses. Empathy towards animals includes the ability to recognise and understand the emotions of animals and is associated with both more positive attitudes towards animals and greater sensitivity to the perception of animal pain [47–49]. This element of teaching regarding equid sentience in high intervention communities may have influenced the higher proportion of owners from these areas believing that their equids could feel emotions and pain. Unexpectedly however, attitudes to animal emotion and pain were significantly different in the high and low intervention groups with the no intervention group intermediate. This may be because other factors (such as community differences) may have played a role in addition to intervention level. Therefore, further research is needed to confirm this link. It may be expected that differences in lameness would be seen in areas where farriery courses had been implemented, however there were no significant difference in lameness between intervention levels. This may be due to the low general amount of lameness seen across the study communities. An in depth examination of hoof condition and balance such as that used in [23] would have been needed to investigate differences in hoof health between intervention levels, but this was not possible in this study. It is also likely that the course participants gained skills outside of the scope of the specific training, for example it has been demonstrated that handling workshops improve owner ability to carry out routine management practices such as lifting limbs which enable hoof trimming [22]. Approach of equids in an appropriate, anxiety reducing manner is also taught and modelled by course instructors as part of the farriery course. It was demonstrated that The Donkey Sanctuary staff can additionally act as a point of future contact, with some previous course participants stating that if they have a health or welfare concern they contact a member of The Donkey Sanctuary staff directly. Future research in this area would also benefit from evaluating welfare in communities with a wider range of interventions and where interventions have not been implemented for an extended period to investigate whether differences in welfare persist over a long time scale.

In this study there were clear differences seen in the level of social knowledge transfer between not only high and low intervention groups but also between low and no intervention groups, suggesting that the clinics did have an effect on the social transfer of knowledge but that this effect was additive with educational initiatives. The potential for transfer of knowledge to owners through veterinary clinics is recognised, via conversations focussed on issues such as preventative care and adequate nutrition during interactions with owners [50]. However, a criticism of a purely free clinic approach is that it neglects other aspects of welfare such as behaviour [51]. A combined approach where educational initiatives cover welfare content outside the medical scope of clinic treatment may be more effective in improving equid welfare in a more complete, holistic manner [52]. Our results suggest that the element of education in high intervention areas is linked to welfare over and above those areas that have only received free veterinary clinics. This, suggests that the practices learned by individuals attending educational workshops, courses and clinics may be being transferred more widely within the community, resulting in better overall standards of welfare. The differences seen in owner attitudes, regarding the belief that their equid could feel emotions and pain, suggest that the knowledge learned may also be creating long term attitude change within communities, although results are mixed. Equid owners who had attended the handling or farriery workshops were frequently those singled out by others as being good with equids. This demonstrates the positive impact that skill introduction can have on the wider community. However, the community structure is an important consideration in the social transfer of welfare information. It has been demonstrated that specific individuals are influential in the acceptance of new practices [30,53]. Key figures such as community leaders and well respected individuals can act as disseminators of information within a community; therefore their identification and inclusion in any initiative is key to ensuring that the mechanism of social transfer of that knowledge is as effective as possible [26,32]. Social cohesion has been seen to affect the motivation and willingness of an individual to invest their time and effort in sharing knowledge with others [54]. Therefore a cohesive community is fundamental in allowing the effective social transfer of welfare information. Research into ‘hard wins’ (the circumstances under which improvement of equid welfare is very difficult) identified a lack of community cohesion and deep-seated social issues as being factors affecting the failure of welfare initiatives despite extensive resource application [55]. This could be a potential barrier to the suitability of initiatives relying on the social transmission of introduced information, previously highlighted in reference to city areas [51]. It may also lead to systematic variation in the choice of communities that NGOs work with, and warrants further exploration.

In conclusion, better welfare (in particular body condition and skin alteration scores) was seen in communities where a history of combined free veterinary treatment and educational initiatives had taken place. Levels of the social transfer of welfare knowledge were also higher in these communities, suggesting that the discussion and transfer of equid welfare knowledge within communities can act as a mechanism to disseminate good welfare practices more widely within a community. The use of a combined approach may enhance the success of future welfare initiatives although the structure and dynamic of the target community should be taken into account.
